# Comparative study on twinning characteristics during two post-weld compression paths and their effects on joint enhancement

**DOI:** 10.1038/srep39779

**Published:** 2016-12-23

**Authors:** Zhe Liu, Renlong Xin, Dongrong Li, Liyun Sun, Qing Liu

**Affiliations:** 1 College of Materials Science and Engineering, Chongqing University, Chongqing, China; 2 National Engineering Research Centre for Magnesium Alloys, Chongqing University, Chongqing, China

## Abstract

Friction stir welding (FSW) has promising application potential in Mg alloys. However, the texture distribution in stir zone (SZ) is usually complicated for Mg alloys, which deterioriates the joint performance. In this study, the texture distribution in SZ was tailored by applying two kinds of post-weld compression deformation along normal direction (ND) or welding direction (WD) of the FSWed AZ31 Mg alloy plates. The twinning behavior and texture change in the various regions of SZ were then evaluated by electron back scatter diffraction (EBSD) characterization. The effect of texture change on the joint performance was discussed in terms of Schmid factors (SFs) for basal slip and extension twinning. The results showed that profuse extension twins were formed through the whole SZ for the sample subjected to compression along ND, whereas they were observed mainly in SZ-side for the sample compressed along WD. Most of the twins were present in the forms of twin bands or chains. The directions of the twin bands or chains were related to the habit plane traces of selected twin variants. The ND post-weld compression had better strengthening effects on the joints compared to the WD compression, and the underline mechanism was discussed.

Friction stir welding (FSW) is a solid state joining method, which has promising application potential in Mg alloys^1^,^2^,^3^,^4^,^5^. Extensive studies have been conducted on various series of Mg alloys subjected to FSW. Most of previous studies aimed to understand the effects of different welding parameters on the microstructure and texture evolutions and dynamic recrystallization and precipitation behaviors in welded materials^6^,^7^,^8^,^9^,^10^,^11^,^12^,^13^,^14^,^15^,^16^. An increasing number of studies indicated that the texture distribution in friction stirred zone (SZ) of Mg alloys is complicated, being a critical factor for the joint performance^6^,^7^,^17^,^18^,^19^,^20^,^21^,^22^,^23^. FSW Mg joints frequently broke in SZ close to the SZ/transition zone (TZ) interface (termed as SZ-side) due to texture softening induced strain localization, as reported in literature^18^,^24^,^25^,^26^,^27^,^28^,^29^. Therefore, texture control during or after FSW is important for optimizing the mechanical properties of Mg joints.

The texture distributions in SZ of Mg alloys can be optimized by adjusting FSW parameters^6^,^7^,^30^,^31^,^32^,^33^,^34^. However, the extent of texture redistribution was usually limited by this method. Recent studies indicated that the FSW texture in Mg alloys could be easily re-distributed by applying post-weld plastic deformation^20^,^35^,^36^. In fact, such a strengthening strategy has been widely investigated on Mg alloys in the form of rolling sheets or extrusion rods^37^,^38^. Generally, the strengthening effect was significant owning to the drastic texture change caused by 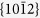 extension twinning deformation. Recently, the effects of twinning deformation on texture variation and strength enhancement have been reported in FSW joints of AZ31 Mg alloys^35^,^36^. The twinning deformation they applied includes post-welding rolling and tensile deformation. However, so far reports on the effect of post-welding deformation on FSW joint performance are still limited.

As known, each twinning has six crystallographically equivalent variants. The texture component produced by twin lamellae is closely related to variant selection, and the selection of variants is dependent on grain orientations with respect to the loading direction^39^. Therefore, loading direction will largely affect the characteristics of twin lamellae in SZ of Mg alloys. In addition, it was reported that grain orientations varied significantly from SZ-center to SZ-side^18^,^24^,^25^,^29^,^40^. Thus, the activation preference, variant selection of twins and their effects on strength enhancement will be different at the various regions of SZ. These issues have not been well understood before.

In this study, uniaxial compression was applied on AZ31 FSW joints along two different directions. The characteristics of the twin lamellae introduced and their effects on texture redistribution were examined in the various regions of SZ by electron backscatter diffraction (EBSD) techniques. Then, the strengthening effects of texture redistribution on two major deformation modes were comparatively evaluated in terms of Schmid factor (SF). The general features of twin lamellae in the two compression processes and their strengthening mechanisms on Mg FSW joints were obtained.

## Results

### Initial microstructure and texture

**Figure 1:**
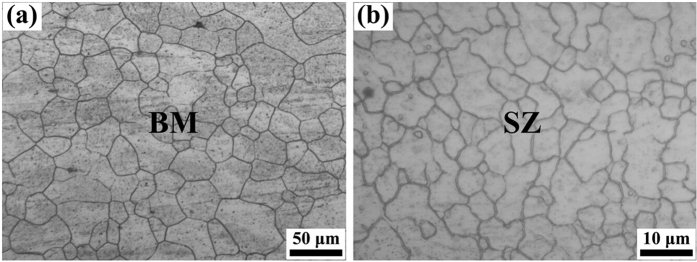
Microstructure of the FSW AZ31 Mg alloy: (**a**) BM and (**b**) SZ.

The typical microstructures of BM and SZ are shown in Fig. 1 using OM. The grains in both samples are nearly uniform. The grain size is determined by a linear intercept method, which is 30~45 μm in BM and 8~12 μm in SZ. Obviously, the microstructure was significantly refined after FSW. This is one major advantage for applying the FSW method in Mg alloys^41^,^42^. The great refinement of grains is a consequence of complete recrystallization caused by the severe plastic deformation and frictional heat during the welding process.

**Figure 2:**
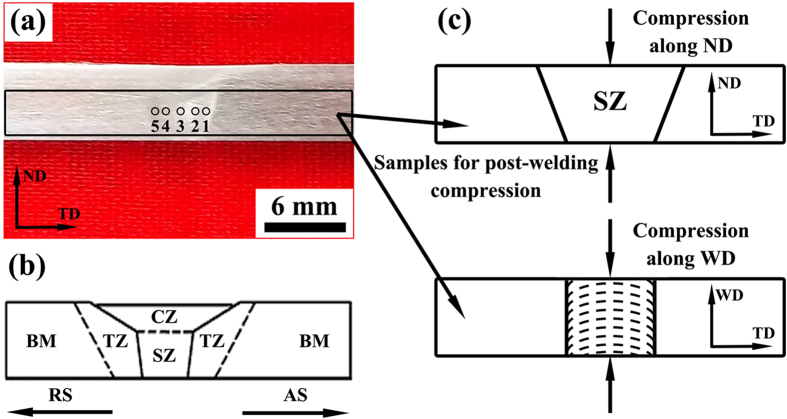
(**a**) Macrograph of cross-section of the FSW AZ31 alloy and (**b**) the corresponding zones; (**c**) schematic for the two post-weld compressions.

**Figure 3:**
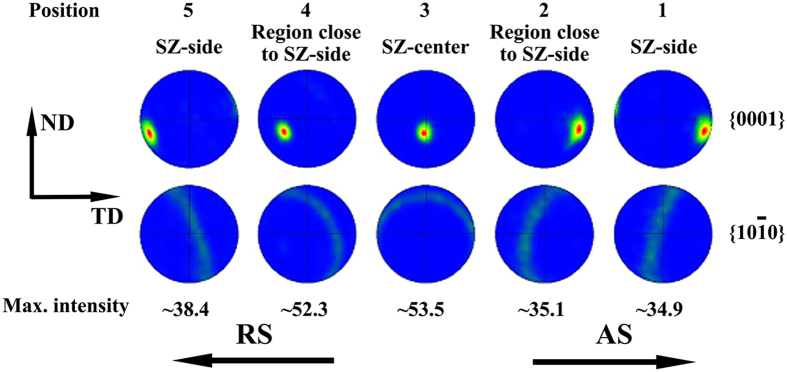
Pole figures in various regions of the FSW alloy.

The as-received AZ31 alloy rolled sheet has a typical basal texture. After FSW, the micro-texture in regions 1–5 of Fig. 2a was examined and displayed in Fig. 3. Typical characteristic of texture distribution was revealed, that is, the c-axis tends to tilt from TD to WD with the position moving from SZ-side to SZ-center. Moreover, the c-axis is tilted away from the TD-WD plane through SZ due probably to the tool tilt angle applied during FSW. The regions 2 and 4 are commonly referred to as the region close to SZ-side. The grains in these regions are favorable for basal slip during transverse tensile tests. In the following, the texture change in regions 2 and 4 will also be focused in addition to SZ-side and SZ-center.

### Microstructure and texture change after post-weld compression

**Figure 4:**
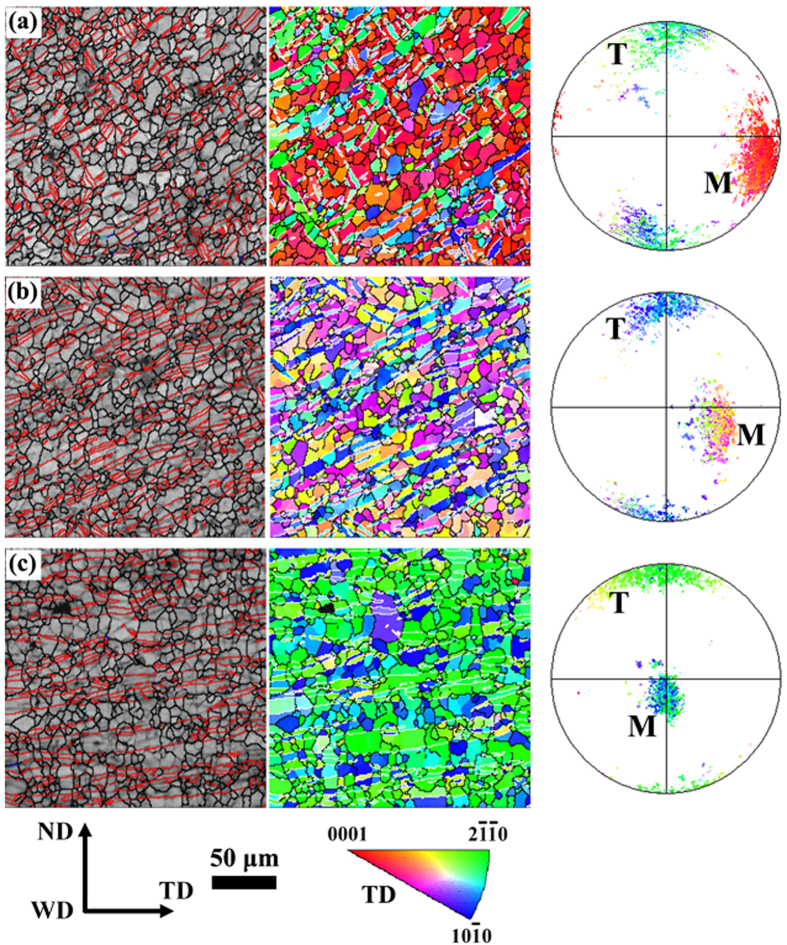
EBSD maps and (0001) pole figures in various regions of the NDCP4 sample: (**a**) SZ-side, (**b**) the region close to SZ-side and (**c**) SZ-center.

The EBSD maps of NDCP4 sample are displayed in Fig. 4 and their texture changes are revealed in the corresponding (0001) pole figures on the right side of the maps. On the left band contrast maps, red lines denote the extension twin boundaries with 5° tolerance. It is evident that profuse twinning was occurred in the whole SZ of NDCP4 sample examined. The (0001) pole figures indicate that new texture component was formed in addition to the matrix texture due to twinning. Although the matrix grains have different texture in the various regions, the twins tend to rotate to the similar orientation, i.e., ND.

**Figure 5:**
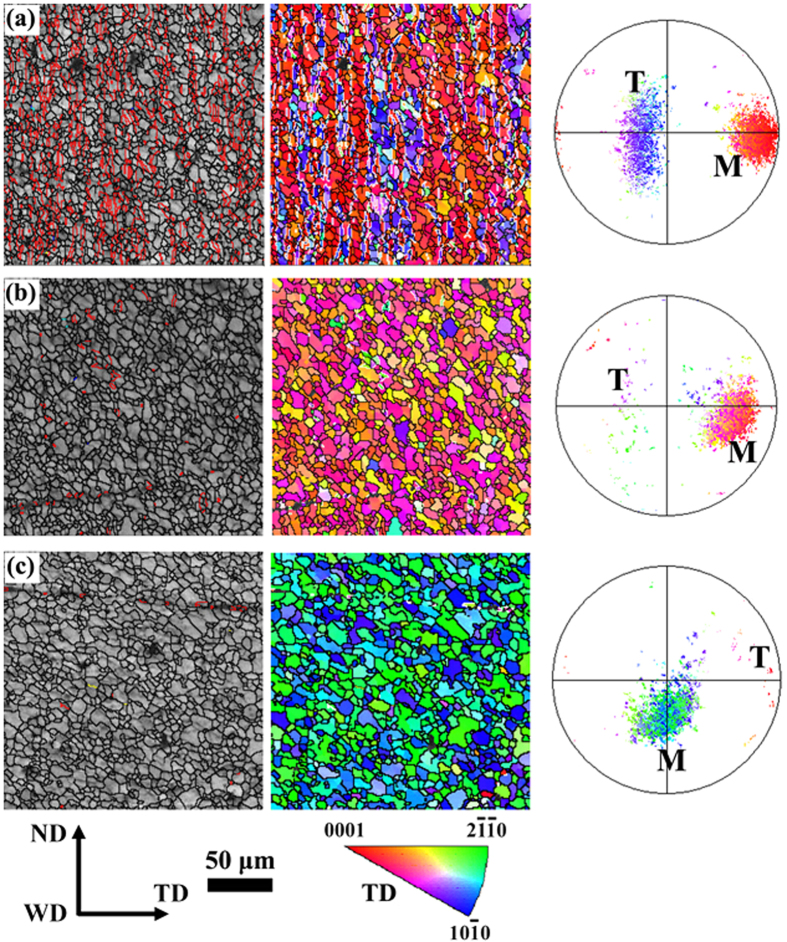
EBSD maps and (0001) pole figures in various regions of the WDCP4 sample: (**a**) SZ-side, (**b**) the region close to SZ-side and (**c**) SZ-center.

Similarly, the evolution of microstructure and texture for WDCP4 sample are presented in Fig. 5. A number of 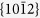 twins were also found in SZ-side. However, little twins were occurred in the region close to SZ-side and SZ-center. This is the major difference from that in NDCP4 sample. In addition, the directions of twin bands formed in Fig. 5a are different from that shown in Fig. 4. The twin bands in NDCP4 sample are directional ~34° or ~56° in SZ-side, ~30° in the region close to SZ-side and ~0° in SZ-center with respect to the horizontal direction (i.e. TD). However, most twin bands in SZ-side are directed almost parallel to the vertical direction (i.e. ND) in WDCP4 sample. In addition, the twins in Fig. 5a have different orientations compared to that in Fig. 4a though the matrix texture is the same. The reason for this will be discussed in detail later.

### Mechanical properties

**Figure 6:**
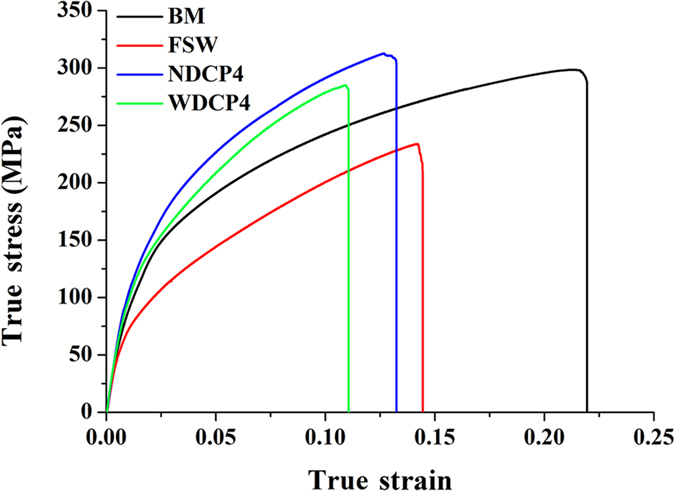
The stress-strain curves of various samples for tension along TD at room temperature.

Figure 6 show the stress-strain curves of the samples, including BM, FSW, NDCP4 and WDCP4 samples. The detailed mechanical properties can be extracted from the curves and are listed in Table 1. It confirms that the tensile properties were largely declined after FSW. Specifically, the decrease in yield strength (YS) and ultimate tensile strength (UTS) is 35 and 64 MPa, respectively. The decline is attributed to the texture softening with respect to basal slip and extension twinning in the welded region, which will be explained in detail later. The elongation is also largely reduced in FSW sample compared to BM. This is attributed primarily to the inhomogeneous deformation ability in the different regions of weld zone. After the tensile tests, the plastic deformation was mainly localized in the region near the interface between SZ and BM. This phenomenon has been explained well in previous studies^25^,^26^,^27^,^28^,^29^ and will not be detailed here.

After some post-welding deformation, twin lamellae were introduced in everywhere of the welded region in NDCP4 sample, as shown in Fig. 4. They were also produced in SZ-side of WDCP4 sample, as shown in Fig. 5. Correspondingly, the texture distributions in FSW sample were modified by the profuse twinning deformation. It is known that the mechanical properties of Mg alloys are largely dependent on grain orientations (i.e. texture) with respect to the loading direction^43^,^44^. Therefore, the texture modification by the introduction of twin lamellae will tailor the mechanical properties. As shown in Table 1, the YS are significantly enhanced in NDCP4 and WDCP4 samples. The increment is by 84.6% and 63.1% for the former and the latter, respectively as compared to FSW sample. The UTS is also largely increased after the post-welding compression; the increment is 78 and 51 MPa for NDCP4 and WDCP4 samples, respectively. It is interesting that the post-welding compression along ND and WD had different hardening effects on FSW sample. NDCP4 sample obviously exhibits better mechanical properties than WDCP4 sample. The YS (UTS) is 14 (27) MPa larger in the former than the latter. This is associated with the different amount of twin lamellae introduced in the FSW samples during the post-welding compression. As shown in Figs 4 and 5, much more twin lamellae were produced in NDCP4 and WDCP4 samples. The texture change induced by twin lamellae will cause different hardening effects on basal slip and extension twinning. This issue will be discussed later in terms of SF analysis. In addition, Table 1 reveals that the ductility is not declined much compared to FSW samples. This is one advantage of strengthening Mg alloys by twin lamellae as reported before^37^,^38^.

## Discussion

### Twin variant selection and its effect on twin morphology

**Figure 7:**
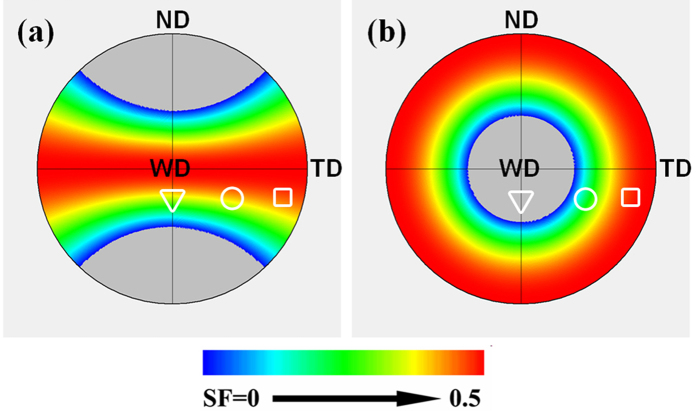
Calculated SFs for 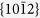 twinning as a function of (0001) poles for (**a**) ND compression and (**b**) WD compression. The major textures in SZ-side, the region close to SZ-side and SZ-center of the FSW sample are superimposed on the pole figures and are indicated by □, ○ and ∇, respectively.

It is generally accepted that the activation of 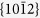 twinning is closely related to SFs^45^,^46^. To understand the difference of twin activation in the various regions of NDCP4 and WDCP4 samples, the distribution of SFs for extension twinning were calculated as a function of grain orientations. The calculation was based on a compression stress applied along ND or WD. The results were displayed in the (0001) pole figure as shown in Fig. 7. The positions of texture peaks in SZ-center, the region close to SZ-side and SZ-side are superimposed on the calculated (0001) pole figures. It should be mentioned that each point in the (0001) pole figure actually represents a set of orientations sharing a common <0001> direction, and there are six possible variants for each twinning. During the theoretical calculations, all the variants and orientations have been considered. However, for the sake of clarity, only the variant and orientation with the largest SF was presented in the pole figures. Figure 7 indicates that all the grains in NDCP4 sample are favorable for twinning because they are located in a high SF region. However, for WDCP4 sample, only SZ-side has high twin SF; SZ-center has negative twin SF (indicated by grey color). Here, a negative SF means that the corresponding twin shear generated an opposite strain with respect to the macroscopic applied strain. This is consistent with the observation shown in Fig. 5. The region close to SZ-side has medium SF values. However, considering the grains have nearly 45° with WD, they are very favorable for basal slip. Therefore, twins were not easily activated there.

**Figure 8:**
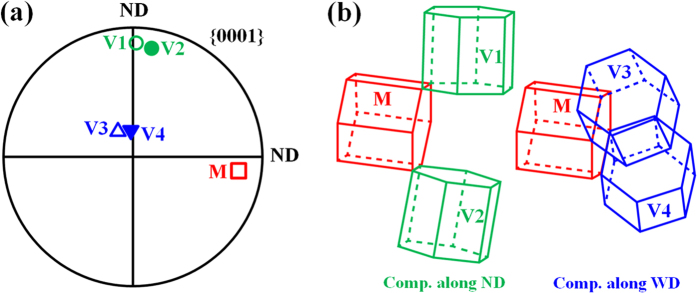
(**a**) (0001) pole figure showing one representative grain orientation of (82.4° 79.3° 8.4°) in SZ-side; (**b**) schematic of the predicted twin variants with the first and second highest SFs in this grain for compression along ND or WD. The orientations of these predicted twin variants are also shown in the pole figure.

Although the SZ-side region is favorable for twinning in both NDCP4 sample and WDCP4 sample, the twins were rotated to distinct orientations. This is probably attributed to the selection of different twin variants. To demonstrate such an effect, Fig. 8a displays a typical matrix orientation (82.4° 79.3° 8.4°) in SZ-side. The SF values of the six possible twin variants can then be calculated under the applied compression along ND or WD. Then the variants with the first and second highest SFs can be determined, and the orientations of these variants were illustrated in Fig. 8b. The results show that variants V1 and V2 have the best activation preference in NDCP4 sample, while variants V3 and V4 do in WDCP4 sample. The texture components attributed to the different twin variants are displayed in the (0001) pole figure in Fig. 8a. It is clear that the selection of the preferred variants in NDCP4 and WDCP4 samples causes distinct texture evolution, which is in agreement with that shown in Figs 4a and 5a.

**Figure 9:**
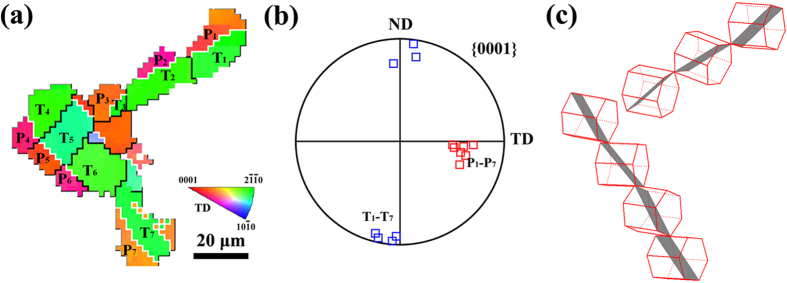
(**a**) EBSD map of twin bands in SZ-side of NDCP4 sample; (**b**) crystallographic orientation of the parent grains and twin lamellae; (**c**) schematic of the grain orientations of P1-P7 (from top to down) and the corresponding twinning planes.

As shown in Figs 4 and 5, the active twins likely formed as twin bands and were oriented in different directions in the various regions. To understand the underline mechanism, twin plane traces of two typical twin bands in SZ-side of NDCP4 sample were shown in Fig. 9. It is confirmed that all the twinned grains have close orientations and the twins were rotated to similar orientations near ND. By a crystallographic analysis, the active twin planes can be determined and their orientations are illustrated by unit cells in Fig. 9c. It is evident that all the active twin planes in the same twin band are well aligned. The directions of the twin bands are approximately parallel to the traces of the active twin planes. Figure 9c also indicates that the variants between the different twin bands are likely of co-zone variants. In fact, the co-zone variants have very similar SF values under compression. Therefore, they have equal preference to be activated. However, the twins in the same bands prefer to have close twin plane normal and shear directions. This is confirmed by the fact that the every connected twin in Fig. 9a has a high geometric compatibility factor larger than 0.9. The definition and calculation method for the geometric compatibility factor can be found from previous studies^47^,^48^. Similar analysis was performed on twins bands found in SZ-side of WDPC4 sample. It consistently revealed that the direction of twin bands is nearly parallel to the traces of the active twin planes. The above analysis implies that such twin bands might be formed via a twin shear transfer mechanism because of the good alignments of twin systems between neighboring grains.

### Strengthening effect of the introduced twin lamellae

**Figure 10:**
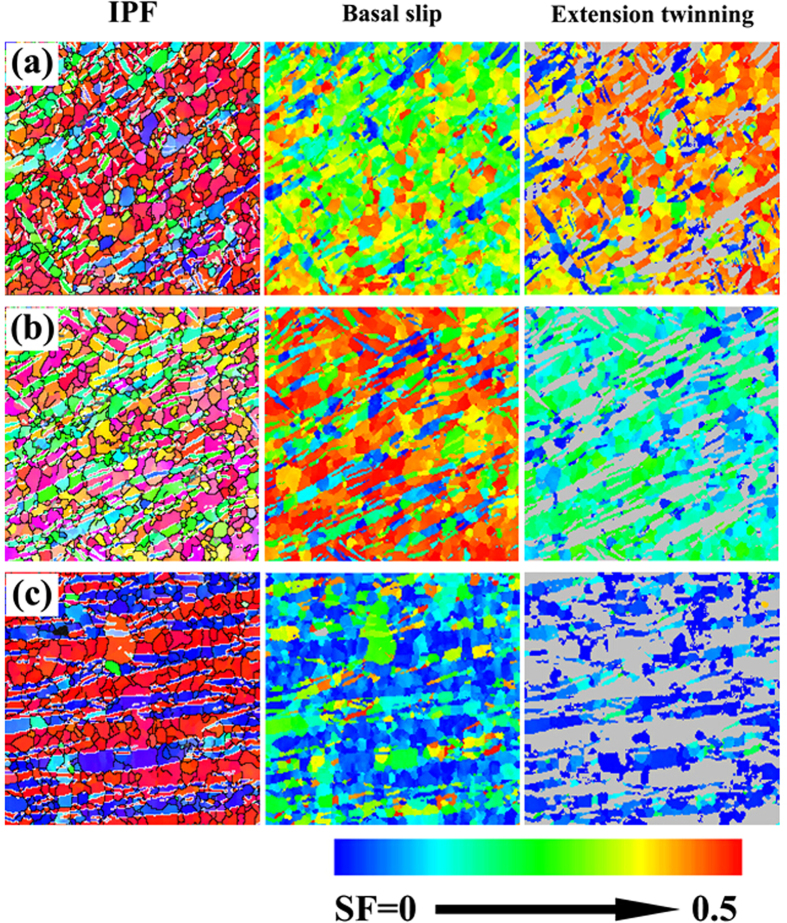
Orientation maps and SF maps in different regions of NDCP4 sample: (**a**) SZ-side, (**b**) the region close to SZ-side and (**c**) SZ-center.

To understand the influence of twin bands on the transverse tensile strength of FSW samples, Fig. 10 show the SF maps of NDCP4 sample. The SF values were calculated based on a tension stress applied along TD. During the calculations, all the six possible variants have been considered. However, for simplicity, only the variant with the largest SF was presented in the SF maps. Moreover, an appropriate color code was used in the IPF maps to distinguish the twin bands from the parent grains. It is evident that the introduction of twin bands does not change much the SF for basal slip in SZ-side, while it has significant strengthening effects on twinning. For the region close to SZ-side, the presence of twin bands largely decreases the SF values for both basal slip and extension twinning. The SF values for both basal slip and extension twinning are relatively small in SZ-center, and the introduction of twin bands does not have much influence. The SF maps of WDCP4 sample are also obtained and analyzed (not shown here), which confirms the similar strengthening effects of twin bands on twinning in SZ-side compared to NDCP4 sample.

**Figure 11:**
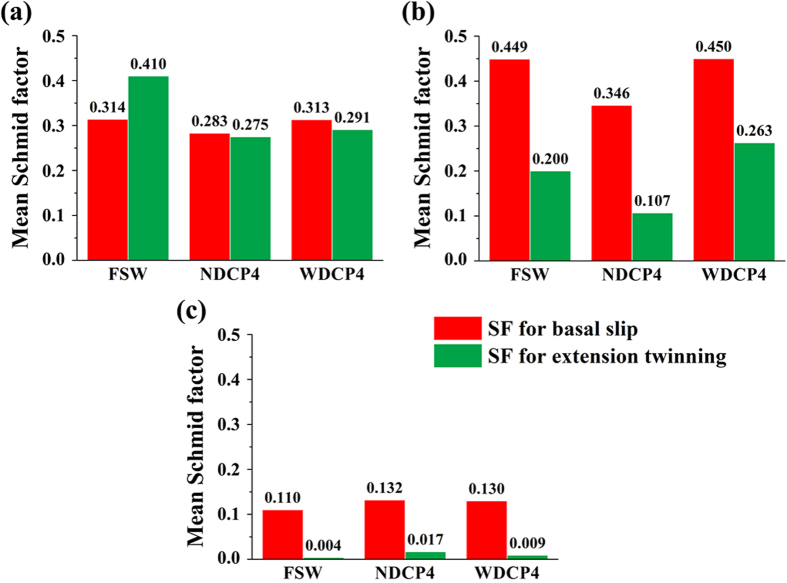
Mean SFs for basal slip and extension twinning in (**a**) SZ-side, (**b**) the region close to SZ-side and (**c**) SZ-center.

The mean SF values for the areas of twins and matrices were calculated and their values were then compared with the mean SFs of the FSW sample without post-weld compression. Figure 11 displays the calculated results in various regions of FSW, NDCP4 and WDCP4 samples, and for comparison purpose, each region from different samples were displayed in separate figures. In SZ-side (Fig. 11a), both the mean SFs for basal slip and extension twinning decrease to a similar level after post-welding compression along ND and WD and the effect of the post-welding compression on extension twinning is likely to be much more pronounced compared to basal slip. Thus, an evident hardening on YS is observed in the post-welding compression samples. For the region close to SZ-side, obvious decrease was noted in SFs for both basal slip and extension twinning for NDCP4 sample. The SF change for basal slip is negligible for WDCP4 sample (Fig. 11b), while the SF for extension twinning increases slightly. This slight SF increase may not cause obvious softening compared to FSW because basal slip instead of extension twinning is the major deformation mode in this region. However, it implies that the hardening on basal slip and extension twinning is less effective in WDCP4 sample as compared to NDCP4 sample in terms of the SF change. This is probably the major reason why NDCP4 sample has higher YS and UTS than WDCP4 sample (see Fig. 6 and Table 1). In the case of SZ-center, the mean SFs for basal slip and extension twinning remained nearly unchanged in the three types of samples, confirming that SZ-center was always the hard orientation irrespective they were subjected to ND or WD compression.

The introduction of twin lamellae in weld zone reorient the crystal lattice, which reduces Schmid factors for the most easily activated deformation mechanisms and hence obviously improve the YS. Furthermore, the presence of twin lamellae retards dislocation slip, causing Hall-Petch type strain hardening. As shown in Fig. 6, the post-deformed samples have stronger strain hardening than the initial FSW sample, confirming the above speculation. Figure 11b confirms that the strengthening in the region close to SZ-side is not effective for WDCP4 sample compared to NDCP4 sample. This is one reason for the fact that NDCP4 sample has higher UTS than WDCP4 sample. Although the YS and UTS are significantly improved for NDCP4 and WDCP4 samples compared to FSW sample, their Els are slightly decreased (see Fig. 6). One possible reason is given below. During the post-deformation, a lot of dislocations were generated in NDCP4 and WDCP4 samples. The presence of dislocations contributes to YS and UTS, but it is not good for El. Moreover, during the transverse tests, slip interaction and slip-twin interaction may occur at the pre-existing twin boundaries, which may cause fracture and reduce El.

## Conclusions

The microstructure and texture changes during post-welding compressin were comparatively studied between two different strain paths, i.e., compression along ND and WD. Their effects on the joint performance were evaluated and analyzed in tersm of SF changes for basal slip and extension twinning. The main conclusions were drawn as follows:

1. For NDCP4 sample, extension twins were formed through the whole SZ. Although the matrix texture is different among SZ-center, SZ-side and region close to SZ-side, the twin texture tended to concentrate around ND.
2. For WDCP4 sample, profuse extension twinning was only occurred in SZ-side, which produced a twin texture around WD. The different twinning propensity between NDCP4 sample and WDCP4 sample was in accord to SF calculations.
3. The majority of extension twins occurred in the form of twin bands or chains in NDCP4 sample and WDCP4 sample. The directions of the twin bands or chains are related to the habit plane traces of the selected twin variants.
4. NDCP4 sample had better transverse mechanical properties than WDCP4 sample. The effects of the introduced twin lamellae on mechanical properties were assessed by comparing the SF variations for basal slip and extension twinning in different SZ areas, which explained the experimental observation.

## Methods

A hot-rolled AZ31 (Mg-3%Al-1%Zn) alloy sheet were used as the starting material. Base materials (BM) with dimensions of 300 mm (WD) × 110 mm (TD) × 6 mm (ND) were machined from the Mg alloy sheet for subsequent FSW experiment. Here, WD, TD and ND refer to the welding direction, transverse direction and normal direction, respectively. FSW was conducted with the tool tilt angle of ~2.5° at a rotation rate of 1600 rpm and a welding speed of 600 mm/min. Before welding, the plates were polished by abrasive paper and cleaned with acetone. A cylindrical thread pin tool with a probe length of 5.7 mm, a pin diameter of 5 mm and a shoulder diameter of 15 mm was used in the experiment.

Microstructure and texture examinations were conducted by optical microscopy (OM) and EBSD. The EBSD detector used was an HKL Channel 5 system (Oxford Instruments) placed in a field-emission gun scanning electron microscope (SEM). The cross-sections vertical to WD of the FSW joint were examined. The metallographic specimens were prepared by mechanical polishing and were etched with a solution consisting of 2 ml distilled water, 2 ml glacial acetic acid, 14 ml ethanol and 0.84 g picric acid. The samples for EBSD analysis were prepared by electrochemical polishing with commercial AC2 electrolyte at 20 V and 20 °C. The step size of EBSD scanning is 1 μm.

Figure 2a shows an overview of the FSW sample. As schematic in Fig. 2b, the whole sample can be divided into the following regions: crown zone (CZ), SZ, TZ and BM, which is typical in FSW Mg alloys^25^,^26^,^49^. The preparation for the post-welding compressed samples is schematic in Fig. 2a. As reported before, the surface layer generally had complicated texture due to the compressive stress from the stir pin and tool shoulder^4^,^40^,^49^,^50^. To simplify the analysis and discussion, the crown zone was excluded in the post-welding compressed samples. The post-welding compression was conducted along ND or WD to a pre-set strain level (~4%) at room temperature. Hereafter, the samples compressed along ND and WD were termed as NDCP4 and WDCP4, respectively (see Fig. 2c).

To evaluate the strength enhancement, tensile tests were performed on the as-received plate, initial joint and post-welding compressed specimens along TD. The specimens for tensile tests have a gage dimension of 30 mm in length, 10 mm in width and 2 mm in thickness, and were grinded by abrasive paper to get smooth surface. The tensile tests were performed at a strain rate of 1 × 10^−3^ s^−1^ at room temperature.

## Additional Information

**How to cite this article**: Liu, Z. *et al*. Comparative study on twinning characteristics during two post-weld compression paths and their effects on joint enhancement. *Sci. Rep.* **6**, 39779; doi: 10.1038/srep39779 (2016).

**Publisher's note:** Springer Nature remains neutral with regard to jurisdictional claims in published maps and institutional affiliations.

## Figures and Tables

**Table 1:** Transverse tensile properties of the various samples.

Materials	YS (MPa)	UTS (MPa)	El (%)
BM	100	298	22.6
FSW	65	234	14.4
NDCP4	120	312	13.4
WDCP4	106	285	11.1
